# Study protocol for a group randomized controlled trial of a classroom-based intervention aimed at preventing early risk factors for drug abuse: integrating effectiveness and implementation research

**DOI:** 10.1186/1748-5908-4-56

**Published:** 2009-09-02

**Authors:** Jeanne Poduska, Sheppard Kellam, C Hendricks Brown, Carla Ford, Amy Windham, Natalie Keegan, Wei Wang

**Affiliations:** 1 American Institutes for Research, Baltimore, MD, USA; 2 Department of Epidemiology and Public Health, University of Miami, Miller School of Medicine, Miami, Florida, USA; 3 Innovations Institute, Division of Child and Adolescent Psychiatry, University of Maryland, Baltimore, MD, USA; 4 Department of Epidemiology and Biostatistics, College of Public Health, University of South Florida, Tampa, FL, USA

## Abstract

**Background:**

While a number of preventive interventions delivered within schools have shown both short-term and long-term impact in epidemiologically based randomized field trials, programs are not often sustained with high-quality implementation over time. This study was designed to support two purposes. The first purpose was to test the effectiveness of a universal classroom-based intervention, the Whole Day First Grade Program (WD), aimed at two early antecedents to drug abuse and other problem behaviors, namely, aggressive, disruptive behavior and poor academic achievement. The second purpose--the focus of this paper--was to examine the utility of a multilevel structure to support high levels of implementation during the effectiveness trial, to sustain WD practices across additional years, and to train additional teachers in WD practices.

**Methods:**

The WD intervention integrated three components, each previously tested separately: classroom behavior management; instruction, specifically reading; and family-classroom partnerships around behavior and learning. Teachers and students in 12 schools were randomly assigned to receive either the WD intervention or the standard first-grade program of the school system (SC). Three consecutive cohorts of first graders were randomized within schools to WD or SC classrooms and followed through the end of third grade to test the effectiveness of the WD intervention. Teacher practices were assessed over three years to examine the utility of the multilevel structure to support sustainability and scaling-up.

**Discussion:**

The design employed in this trial appears to have considerable utility to provide data on WD effectiveness and to inform the field with regard to structures required to move evidence-based programs into practice.

**Trial Registration:**

**Clinical Trials Registration Number**: NCT00257088

## Background

The educational sector, as a normative setting for children, is an important delivery system for drug abuse prevention. A number of preventive interventions directed at aggressive, disruptive behavior and other antecedent risk factors such as poor achievement have shown both short-term and long-term impact in epidemiologically-based randomized field trials. However, prevention programs are often not implemented with high quality in schools [1-3]. Until recently, the primary concern of prevention researchers has been to test the impact of interventions through efficacy and effectiveness trials. The result is that many interventions have been tested without precise specification of the model of support necessary to implement and sustain intervention practices with high quality over time.

In fall 2003, we began the third randomized field trial carried out by the senior members of this research team in collaboration with the Baltimore City Public School System. This trial focused on testing interventions aimed at aggressive, disruptive behavior and poor achievement, separately and in combination. Results of the first two trials [4-15] provided support for undertaking this trial in which we combined three intervention components--classroom-behavior management; academic instruction, particularly in reading; and family-classroom partnerships--into one integrated intervention called the Whole Day First Grade Program (WD). This trial was designed to bring together effectiveness and implementation research. The design supported an effectiveness trial of the WD compared with the standard school district program (SC). The design also supported an examination of the utility of a multilevel structure to support high levels of implementation during the effectiveness trial, to sustain WD practices across additional years, and to train additional teachers in WD practices. This paper presents the implementation portion of the protocol in which we followed teachers with subsequent cohorts of children to study sustainability and scaling-up. Also see Additional File 1: 'Description of WD intervention, student sample, and measures of student outcomes' and Additional File 2: 'WD cohort two student sample figure'.

### Specific aims and hypotheses

The specific aim and hypotheses regarding implementation (aim three) follow logically from the aims of the effectiveness trial (aims one and two).

#### Aim one

Model the malleability of developmental paths by evaluating the effectiveness of the WD program, directed at reducing the antecedent risk factors for later substance abuse, comorbid mental and behavioral disorders, and school failure. We hypothesize that students in WD classrooms will exhibit less aggressive, disruptive behavior, more on-task behavior, and gains in student achievement, particularly in reading, compared with their counterparts in SC classrooms over the course of first grade and to the end of third grade.

#### Aim two

Measure the variation in the impact of WD by examining moderating factors at the level of the child and the social contexts of family, classroom and school, peers, and community. In line with the results from our previous trials, we hypothesize that: the impact of WD will be stronger among children who begin first grade with lower readiness and poorer student behavior than their classmates; and the impact of WD will vary as a function of the quality of teacher practices, with improved teacher practices leading to student improvements in behavior and achievement, proximally and through third grade.

#### Aim three

Examine the utility of the support structure: during the effectiveness trial; as teachers implement WD in consecutive cohorts of first graders (sustainability); and as WD practices are taught by school system employees to teachers new to WD (scaling-up). We hypothesize that: the multilevel support structure will result in sustained high levels of WD practices with three consecutive cohorts of first graders; and the multilevel support structure will result in high levels of WD practices with additional teachers when they are trained in WD practices.

## Methods

### Overview of the design

The within-school design involved 12 public elementary schools and two first-grade classrooms within each school. Within each school, three consecutive cohorts of children were randomly assigned to first-grade classrooms as they enrolled. Classrooms/teachers were randomized to intervention condition in the first year, with one teacher assigned to WD and one teacher assigned to SC. Because every school had a WD and a SC classroom, schools served as blocking factors, and comparison of intervention effects could be obtained for each school. This two-level randomized block design allowed us to hold school, family, and community catchment area factors fixed while examining intervention effects at the classroom level, and examine main effects and test hypothesized variations in impact on the basis of variables such as gender, students' individual aggressive behavior, teacher self-efficacy, and classroom levels of aggressive behavior. Random assignment of children to classrooms allowed two classrooms within a school to be comparable at baseline and was extremely efficient in testing the main effect of a classroom intervention [5,6,16,17].

Aware of the possibility of intervention leakage with this classroom-based, within-school design, we implemented procedures that had successfully limited such leakage in the prior trials, such as meeting with principals monthly [16]. In addition, the SC teachers received training and support in WD as part of the design in the third year of the trial. We found no evidence of contamination in the schools with internal controls in either of the first two trials [5,16] or in this trial.

### Procedures for random assignment

We drew upon our prior experience as well as knowledge in the field to develop protocols for the random assignment of both students and teachers. Here, we describe the procedures for random assignment in this multilevel trial. For details regarding the effectiveness arm of the trial in which we followed students over first grade and into third grade, see Additional File 1: 'Description of WD intervention, student sample, and measures of student outcomes' and Additional File 2: 'WD cohort two student sample figure'.

### Schools

**Figure 1:**
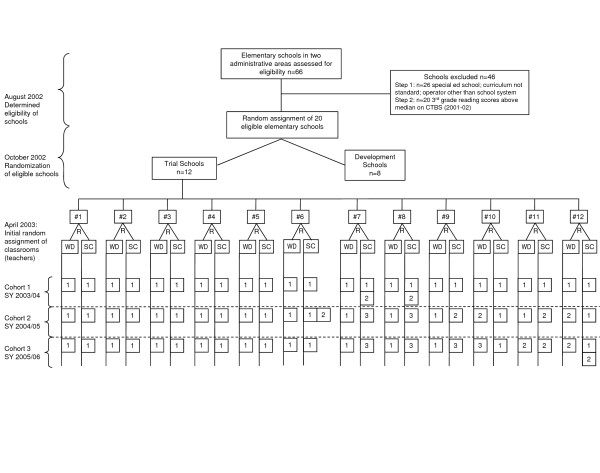
**WD Teachers Over Three Years**. R = random assignment; WD = Whole Day First Grade Program classroom; SC = standard classroom (control); SY = school year.

We began with a pool of 66 elementary schools in two adminstrative areas of the school district. Schools were excluded if all the students attending the school received special education or other special services; the school was operated by an entity other than the school system; the first-grade curriculum was not the standard district curriculum; or the school had fewer than two or more than five first-grade classrooms. We excluded large schools because they were less common and tended to have different organizational structures than smaller schools. Twenty-six of the 66 schools were excluded as a result of these criteria. Because academic achievement was a primary target of the WD intervention, we decided that the schools performing less well academically would be eligible to be part of the WD initiative. The 40 schools remaining after the first exclusion step were ranked by third-grade academic achievement on the standardized achievement test used by the school system, third grade being the lowest grade at which a standardized achievement test was used to rate student achievement. In October 2002, principals of the 20 lowest performing schools participated in a lottery draw to randomly assign schools to participate either as one of eight schools deemed development schools, where we piloted intervention components and conducted staff training on assessment procedures, or as one of 12 schools deemed trial schools, where the randomized field trial would take place (see Figure 1). The trial was conducted in these 12 schools for three consecutive school years beginning in 2003 to 2004.

### Classrooms/teachers

In April 2003, all 37 first-grade classrooms/teachers in the 12 schools participating in the randomized field trial were randomly assigned to condition. In each school, one teacher was randomly assigned to be a WD classroom/teacher, one was randomly assigned to be a wait-listed SC classroom/teacher, and all other first-grade teachers were randomly assigned to be nonparticipating classrooms/teachers. Teachers in both WD and SC classrooms were followed as they taught three consecutive cohorts of first graders. In a type of wait-listed control, SC teachers who served as controls for cohorts one and two were trained in the third year to deliver WD to cohort three first-grade students. The effectiveness trial relied on efficient within-school comparisons of WD and SC for cohort one and cohort two students from first to third grade. The sustainability question centered on whether WD teachers' practices remained high or fell off across the three cohorts. The scale-up question centered on whether the practices of the former SC teachers improved--as well as the consequent child outcomes--when they were trained to deliver WD.

While each of the 12 schools maintained the design by having one WD classroom and one SC classroom each year for three years, there were changes at the teacher level as teachers left the school. We anticipated that some teachers would change over the years, and we established a protocol for such changes. We stipulated that if a teacher left, the classroom would not change condition and the new teacher would be assigned the departing teacher's intervention status. If the replacement was a teacher already in the school, our protocol stipulated that this teacher could not have previously been assigned to a condition (WD or SC) within the trial. The patterns of teacher mobility are shown in Figure 1. There were no changes in either WD or SC teachers in schools one to five; the same teachers taught the WD and SC classrooms for all three years of the trial. Schools six to 12 experienced some degree of teacher mobility.

Overall, the changes were typical of staffing in large urban school districts and a reality when conducting research in real-world settings. For example, in schools seven and eight, the original SC teacher stopped teaching during the first year of the study and a long-term substitute taught for the remainder of the year. In the second year of the study, these classrooms were assigned a permanent teacher who taught first grade in both the second and third years of the study. Of note is the fact that in the second year, school six had two SC classrooms. After the initial assignment of students to classrooms, the school decided to add a third first-grade classroom because enrollment was greater than anticipated. Because we did not have an established protocol for the situation, we worked with the school to determine appropriate actions to maintain the random design. We randomly selected 10 students to come out of the original WD and SC classrooms before the intervention began, creating a new control classroom. The teacher of this newly formed classroom did not have experience teaching first grade and was not trained in the school system's first-grade curriculum, so the original SC class and the new control classroom were combined in a team-teaching model. By the end of the year, these two classes had separated back into two traditional, discrete classes. We decided to collect data in all three classrooms at each time point throughout the year. As a result, we have one WD classroom and two SC classrooms for school six in the second year of the trial. In the third year, school six had only two first-grade classrooms and they were taught by the initially assigned WD and SC teachers. In spite of this unexpected design modification, we were able to maintain random assignment of children.

Although there were no instances of protocols being broken--no teachers changed their design--it is important to note that SC teachers were replaced at twice the rate over the three years of the study (n = 8) compared with WD teachers (n = 4). We have hypothesized that as teachers gain mastery in their classroom with regard to their practices in classroom behavior management and instructional content, they will be less likely to leave a school or the teaching profession. In keeping with this hypothesis, we would expect to see a lower rate of attrition in WD classrooms than in SC classrooms. As we move to an analysis of the data, we will test for systematic bias at the level of the classroom and at the school/community level.

### Structure to support sustainability and scale-up

In developing the support structure required for teachers to learn, implement, and sustain WD practices and for additional teachers to be trained, we focused on three areas: understanding the multilevel organizational structure of the school system; delivering professional development to teachers; and systematically monitoring teacher practices and support to teachers.

### Multilevel structure of the school system

**Figure 2:**
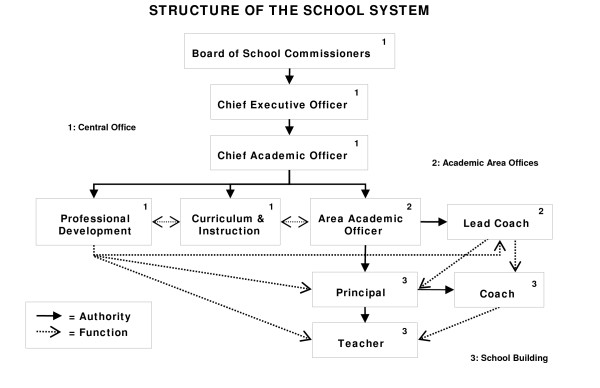
**Multilevel Structure of a School System**.

To understand the level and nature of the mandate, authority, accountability, and resources necessary to sustain and scale-up WD practices, we needed to understand the multilevel organizational structure of the school system. Figure 2 presents the organizational structure at the time of the WD trial. The Board of School Commissioners (the school board) had the legal authority to oversee all operations of the school district. The chief executive officer (CEO) of the school district (the superintendent) oversaw all aspects of school district administration. The chief academic officer (CAO) served under the CEO and was responsible for all K through 12 instruction, academic as well as behavior and social emotional learning. Under the direction of the CAO, the city schools were divided into four elementary areas, a middle school area, and a high school area, each overseen by an area academic officer (AAO). AAOs were responsible for providing support to principals as well as to the schools more generally. Each area office had at least one coach who worked directly with schools to provide professional development. Within the school building, teachers were supported by the principal, school-based instructional coaches, and support staff such as social workers and psychologists.

We worked with the school system to create two teams to support the effectiveness trial and the goal of sustaining and scaling-up practices in the school system--the Core Team and the Schools Committee. The Core Team comprised individuals who held key positions of authority in the school system, along with senior members of the American Institutes for Research (AIR team). District members included the CAO, senior staff from the offices of curriculum and instruction and professional development, and the area superintendents whose schools were participating in the trial. The Core Team was responsible for the implementation and continued monitoring of the randomized field trial within the school district, and met monthly to review progress against benchmarks and to anticipate and resolve problems. The Core Team's role was also to address the challenges that often impede the uptake of interventions into general practice, including the time to deliver the intervention, the ease of implementation, the compatibility of the intervention with the mission and vision of the institution, and the cost of the intervention [1-3,18-29].

Principals of the 12 participating schools, along with Core Team members and senior AIR staff, participated on the Schools Committee. The committee met monthly to address issues pertinent to the field trial, such as maintaining fidelity, developing procedures for randomization of teachers/classrooms and students, engaging with parents and garnering consent, determining ways to introduce the field trial to the school community, maintaining the morale of standard classroom (comparison) teachers, and making decisions at the school level regarding oversight, monitoring, and allocation of resources.

### Professional development to teachers

As part of the WD trial, we addressed the primary concern of school system leaders with regard to professional development for teachers, namely, that the role of the WD facilitator be specified with precision so that practices would be observable and replicable. Because we were interested in building capacity within the school system, we decided that AIR staff would not train teachers directly but would train school district employees to be WD facilitators who would work directly with teachers. This model was decidedly in contrast to the prior two field trials in which research staff worked directly with classroom teachers.

In developing the model of coaching and support to teachers, we were informed by the emerging literature on professional development for educators and on adult learning. This work highlights the importance of providing opportunities for active learning through observation, meaningful discussion, practice, and reflection [30-32]. Research also suggests that professional development is best conceptualized as an ongoing process rather than a single event, and that professional development activities should be aligned with one's professional work [30,33-35] and incorporated into one's daily professional work [30,36-38].

Literature on adult learning and school reform emphasizes the importance of collective participation of teachers within schools, grouped by grade level, or of principals across schools [39-42]. A concept that became critical to our coaching model and our work with the Schools Committee and the Core Team is that supporting change at the organizational level requires linkages across levels in the system, not just within levels in the system [43,44]. Stated another way, while professional community matters at specific levels such as schools [45], it is critical to create communities of learners within and across the various levels of the organization [46,47]. As defined by Resnick and Glennan, nested learning communities are 'organizations in which all individuals and units are expected to upgrade their capacities continuously in accord with a shared set of instructional principles and strategies. In this design, instructional leadership, coupled with reciprocal accountability between 'layers' of the organization, provide professional learning opportunities specifically geared to the district's vision of instruction' [46].

The support provided to the teacher was primarily technical in that the WD facilitator was considered the expert in WD implementation practices and served as a mentor to the teacher. WD facilitators had expertise in the theory and practice of WD and classroom behavior management more broadly as well as the interpretation and use of data regarding teacher practices and student behavior. WD facilitators spent one day per week in the classroom with each teacher and supported teachers in bringing together the new knowledge of WD learned in the pre-implementation training with their craft knowledge of classroom teaching [48]. The WD facilitators spent most of their time working directly with teachers in classrooms, observing, planning, modeling and mentoring, and providing feedback [48,49].

Observations of teacher practices in the classroom provided the foundation of the WD facilitators' work. The facilitators used the WD implementation checklist each month throughout the entire school year to determine the type, amount, and focus of professional development. The checklist provided information regarding the occurrence and quality of teacher practices on both general practices and specific core elements of each component of WD. Using these data, the WD facilitators prioritized coaching needs and worked with the teacher to create a professional development plan that specified goals, target areas for mentoring, and coaching strategies and activities to be undertaken. WD facilitators supported teachers in WD practices through modeling of practices, guided practice, visits to other classrooms to observe WD implementation, conferences, and joint planning. The ability to work through trust with the teacher was a critical skill for facilitators; not all teachers welcomed a facilitator into their classroom or were open to the support the facilitator offered [50,51].

The extant literature provided little guidance about the pace and focus of coaching over the course of a year. We decided on a predetermined amount of coaching support, with WD facilitators spending additional time with teachers who needed more support. Each facilitator supported four schools, devoting one day per week supporting the WD teacher in each school. WD facilitators were trained during the first year of the trial by intervention team members who were part of the research staff.

### Systematic monitoring of intervention practices and support

In recent years, researchers have recognized that the delivery of intervention practices is usually variable and that this variability affects outcomes [1,16,52-59]. To understand the occurrence and quality of WD practices and the support delivered directly to teachers, we measured teachers' practices with regard to WD implementation as discussed above; facilitators' practices in supporting teachers; and the availability of resources such as materials, planning time, and release time for professional development, particularly those provided by the principal.

### Measures and data collection

Classroom observations of student behavior and teacher practices were conducted three times in first grade: baseline/autumn, mid-year/January, and spring/May. Each observation took four hours over two days, and did not disrupt the classroom activities. Teacher interviews about student behavior were conducted with first-grade teachers at the same three time points. When each cohort reached third grade, its current teacher was interviewed. In fall and spring of first grade and spring of third grade, students were assessed individually in a quiet area of the school on reading and reading-related skills; students were also asked about experiencing symptoms of depression and anxiety; see Additional File 1: 'Description of WD intervention, student sample, and measures of student outcomes'. School records were collected at the end of each school year.

### Measures of teachers' practices

We conducted classroom observations using a variation of teacher observation/student engagement [60]. In this time-sampling schema, a teacher's instructional practice and students' behavior are recorded minute by minute. For a teacher's instructional practice, observers code the instructional format and content of teaching. Eight instruction categories range from whole class to small groups to students working on their own, and include a category for non-reading instruction. Twenty content codes cover four domains: reading comprehension, word work, oral language and writing, and non-reading-related activities. The content codes were extended to include feedback in response to behavior (corrective, praise, punitive, directive). Inter-rater reliabilities of >0.80 are maintained. At the end of each timed observation, observers use the checklist of teacher's practices to rate the instructional and behavior management strategies exhibited during the session. The form parallels the WD implementation checklist and provides an independent measure of program implementation in WD classrooms, as well as information on classroom behavior management and instructional strategies in SC classrooms. Items were added to the teacher interview to assess the strategies that teachers used to engage with families and the level of caregiver response to the strategies.

### Measures of fidelity

The WD implementation checklist provided information about teacher practices along two dimensions, practices that were general to the classroom and practices that were intervention-specific. Thirty-six behavioral indicators precisely defining the core elements of the three intervention components were rated on whether defined practices occurred (yes/no) and with what quality (scale of one to six). A global rating was also given for each of the three intervention components. Measures of family-classroom partnership included attendance sheets for activities to which parents were invited, such as classroom orientation, class meetings, and the family read-alouds. The use of the home-link telephone line, a messaging system for teachers and parents, was monitored. Checklists were developed to rate the degree to which principals and facilitators fulfilled their clearly defined roles related to supporting the teacher and the implementation of the WD program.

### Statistical analyses and power

The analyses for Aim three, sustainability and scaling-up of WD practices, proceed logically from the analyses supporting the effectiveness trial of WD.

### Analyses for Aim one: Malleability of developmental paths

We are carrying out formal growth analyses to evaluate how WD affects the course of reading skills and achievement, aggressive behavior, and depressive symptoms. Intervention effects will be modeled as both mean differences in latent growth trajectories for the slope and differences in the covariance between intercept and slope by intervention group, a type of intervention by baseline interaction [61]. These analyses will include school- and classroom-level clustering. Following this series of multilevel analyses for single repeated measures, we will conduct multilevel growth analyses for first-grade through third-grade outcomes [62] to test for both main effects and interactions involving the intervention with baseline characteristics.

### Analyses for Aim two: Variation in impact

We will also examine whether the intervention affects children differently by using baseline measures of reading skills and aggressive behavior, as well as parent involvement. We have found that additive models [11,15], because they include nonparametric models with smooth changes in impact as a function of baseline, are excellent tools to distinguish the degree of benefit that different children receive from this particular intervention. A second method for examining variation in impact is examining how baseline interacts with intervention condition to affect growth trajectories [61,63-65]. These intervention-by-baseline models extend the univariate models into the growth curve framework, and allow changes in slope to be affected simultaneously by both intervention and baseline. Thus we will examine whether the best growth trajectory improvement from the intervention occurs more for higher- or lower-risk children.

A further set of analyses will be based on growth mixture modeling (GMM) [15,64,66] using Mplus software [62]. GMM allows fitting multiple growth trajectories, for example, early and late starters for aggression, and testing whether the intervention affects these groups differently. We can also carry out growth mixture analyses with a categorical distal outcome as well as time-to-event, or survival, measures (such as time of first suspension). In these general growth mixture models (GGMM) [15,64], parameters of individual growth trajectories, including intercepts, slopes, and higher-order shape parameters, may be influenced by baseline characteristics, intervention status, and time-dependent covariates. GGMM also allows individuals to follow a class of growth trajectories. An important part of this model is that class membership is an unobserved category variable. Nevertheless, like latent class analyses, GGMM permits both predictors and responses to be related to the unobserved class membership. Model selection for GGMM will be based on both the bootstrap likelihood ratio test and the Bayesian Information Criterion [67] with examination of model fit [64,68,69].

### Analyses for Aim three: Sustainability and scaling-up of WD practices

We will examine the extent to which the impact of WD on children's learning and behavior is explained by the level of by the teacher implementation, as well as school-level support, over time. We have successfully examined such implementation measures in the past [16] when we determined that poor implementation of the intervention fully explained the lack of improvement in some schools. Sustainability will be tested by comparing the practices and subsequent child outcomes for the WD teachers over two as well as three years with those of their SC counterparts for the first two years. Scaling-up will be tested by comparing the practices and child developmental outcomes for the original SC teachers in their last year with those in previous years and with those of WD teachers. The difference in SC measures across cohorts assesses this extendibility, and the power is expected to be similar to that for testing the intervention in cohort one because here each teacher serves as his or her own control.

### Statistical power

In cohort one, we expect to have power of 0.79 to test the WD main effect on end-of-first-grade reading when the true effect size is 0.25, compared against total variation in the sample. This calculation is based on specification of multivariate means, variances, and covariances from data from prior trials conducted in Baltimore. The prior studies provide estimates of each source of variation as well (children, classrooms within schools, and schools) [70]. It assumes that individual variation is twice as large as school variation, that classroom variation at the time of randomization was negligible, and that the variation in intervention impact by school is a third of the overall variation, with effect size. Because this earlier trial involved a smaller number of schools (nine instead of 12 schools) and a less comprehensive intervention compared with that in the present trial, we anticipate having higher statistical power than in the previous trial. In that trial, we were able to report significant main effects and interactions [10,16]. The power is nearly identical for third-grade main effect analyses as it is for end-of-first-grade analyses, even when we allow for 20% attrition, primarily from mobility. This loss in power through attrition is generally more than offset by the increase in power from growth curve analysis [61].

For testing whether the intervention improved teacher practices, statistical power is slightly higher, for the same size effect, compared with analyses of child outcomes. This difference occurs because the teacher measures are not subject to the added statistical error arising from child variation. Thus, we expect to have sufficient power to detect moderate size effects in this trial on teacher practices across cohorts. Our hypotheses state that the WD effects on the original WD teachers should increase from cohort one to cohorts two and three. We plan to test this with a two-level linear trend model across cohorts. A test for nonlinear trend (one DF) can also be carried out. The trend tests are expected to have higher power than our original tests of sustainability because there should be stronger improvements of these teachers over the cohorts.

## Discussion

The WD trial was deliberately designed to bring together research on program effectiveness with research on program implementation. Although the model of support to teachers was not experimentally manipulated, we are learning a great deal about the multilevel structure that needs to be in place to ensure that teachers can learn, implement, and sustain evidence-based intervention practices with high quality over time and that support practices being scaled-up. This information will inform the next generation of interventions focused on enhancing program implementation.

## Ethical review

Ethical approval for this trial was obtained by the Institutional Review Board at the American Institutes for Research on July 17, 2002.

## Competing interests

The authors declare that they have no competing interests.

## Authors' contributions

JP, SK, and CHB conceived the study and collaborated throughout. JP served as project director of the trial and drafted this manuscript. CHB prepared all randomizations. CF served as the intervention chief for the study. She oversaw the development and refinement of the intervention components and was responsible for the training and supervision of the WD facilitators. NK worked with CF to develop the classroom-family partnership component and served as a community liaison. AW led the collection of assessments and the data management and served as an analyst for the study. WW provided analytic models for mediation and moderation. All authors have read and approved the final manuscript.

## Supplementary Material

Additional file 1 **Description of WD intervention, student sample, and measures of student outcomes**. Description of WD intervention, student sample, and measures of student outcomes. Click here for file

Additional file 2 **WD cohort 2 student sample**. This figure shows the random assignment and design status of a cohort of students from initial random assignment prior to the start of first grade to the end of the first grade year. WD = students randomized to Whole Day First Grade Program; SC = students randomized to standard classroom (control); Other = students randomized to first grade classrooms not participating in the trial; circles denote students who left a study condition and were reassigned to another study condition (denoted by a triangle). Click here for file
