# Supplementary material for: Study protocol for a group randomized controlled trial of a classroom-based intervention aimed at preventing early risk factors for drug abuse: integrating effectiveness and implementation research
Source: Implement Sci. 2009 Sep 2;4:56. doi: 10.1186/1748-5908-4-56 (PMC2753630; doi:10.1186/1748-5908-4-56)
Supplement: Additional file 1 — Description of WD intervention, student sample, and measures of student outcomes. Description of WD intervention, student sample, and measures of student outcomes. [file 1748-5908-4-56-S1.doc]

Additional File 1: Description of WD intervention, student sample, and measures of student outcomes

Here we provide a description of the Whole Day First Grade Program (WD), details about the student sample and denominator using one cohort of three as an example, and a description of the measures used for student outcomes.

**Intervention Conditions**

The WD program enhances the standard curriculum in the areas of classroom behavior management; academic instruction, particularly reading; and family-classroom partnerships. A description of the SC and WD conditions for each component follows.

***Standard program classrooms (SC)*** Classroom behavior management At the time of this trial, the school system trained all new teachers in a commercially available behavior management program called Classroom Organization and Management Program (COMP) [1-4]; teachers already in service did not receive any training in classroom behavior management. Academic instruction The Baltimore City Public School System adheres to the Voluntary State Curriculum for Maryland, which specifies what students should know and be able to do at the end of each grade. The reading curriculum used at the time of the trial was *Collections for Young Scholars* [5]. Family/classroom partnership During the trial, no standard strategies or practices were in place for teachers to employ at the classroom level to engage and involve caregivers in student learning.

***Whole Day First-Grade Program classrooms (WD****)* Classroom behavior management In addition to the COMP training described above, teachers in WD received training in using the Good Behavior Game (GBG) and integrating the game into the activities of the classroom day. GBG is a group-contingent classroom behavior management strategy that promotes classmate/peer concern for each child’s positive behavior by rewarding teams with below-criterion levels of inappropriate behavior [6]. As tested in Baltimore, GBG is built around four core elements integrating (1) classroom rules, (2) team membership, (3) monitoring of behavior, and (4) positive reinforcement to individuals and the group. *Establishing classroom rules:* The teacher works with students at the beginning of the year to clearly and explicitly specify acceptable and unacceptable behaviors and to ensure that students make the connection between classroom rules and the behaviors that constitute breaking and following the rules. Rules are clearly posted on a wall chart, and students have a small laminated version of the wall chart placed on the corner of their desks. *Assigning team membership:* A core element of GBG is the team structure that gives children who exhibit aggressive, disruptive behavior the opportunity to interact with positive role models while the game is being played. After observing students for several weeks, the teacher divides them into several teams of five to eight students, heterogeneous in behavior and learning, with equal membership by sex. These teacher-determined groups deliberately break up naturally forming peer groups that may be occurring in the classroom in which children with like characteristics tend to group together. *Playing the game and monitoring behavior:*At times students are doing work independent of the teacher, the teacher announces “We will play the Good Behavior Game” and sets a timer for the number of minutes the game will be played. The children work cooperatively to maintain good behavior. When a classroom rule is broken, the teacher identifies the behavior and the rule that has been broken and the team receives a point. At the end of the game, each team that has 4 or fewer points receives a reward. Teams do not compete against one another; instead, team members work cooperatively within their team to support positive, appropriate behavior. Teachers continue to use the game over the course of the school year, extending the time the game is played from 10 minutes three times a week at the beginning of the year to every day for longer times throughout the day. *Providing positive reinforcement to individuals and the group:* GBG uses a group reinforcement strategy in which children reinforce one another to benefit the entire group. When first playing GBG, teachers use tangible rewards, such as trinkets, small toys, and school supplies. As the school year continues, the rewards become more intrinsic and natural to the classroom setting, such as extra time to read during the school day.

Academic instruction Teachers in WD classrooms delivered the same reading curriculum as the teachers in the standard classrooms. The primary strategy used to enhance the reading/language arts curriculum in WD classrooms was “guided reading,” an instructional approach in which the teacher works with a small group of children (typically four to six) who demonstrate similar reading processes and skill levels using leveled readers (i.e., texts representing specified instructional levels). During guided reading, the teacher supports individual children by listening as they read these texts and, when necessary, prompting them to monitor their understanding of the text and to use previously taught reading skills. WD classroom teachers used leveled readers to extend and integrate reading skill development in mathematics, science, and social studies.

Family/classroom partnership The Home Link Program [7] was a dedicated toll-free classroom telephone line with voice mail that facilitated regular communication between teachers and parents. The purpose was to build family-classroom partnerships by helping parents stay connected to the classroom through weekly recordings/messages from the teacher, paired with “*fun and easy*” home learning activities to help parents reinforce at home what children were learning in the classroom. Parents could phone in at any time, day or night, to hear the teacher’s message and to leave the teacher messages in turn. Monthly *Family Read Together* sessions focused on parent-child “partner reading.” The teacher provided guidance *and* support to parents through structured activities that built parents’ knowledge and confidence for carrying out strategies at home to help their children learn to read.

Training and support As described in the study protocol, WD teachers received approximately 50 hours of professional development over and above the training that teachers ordinarily received from the school system. Multiple training formats were used in WD, including workshops, cluster meetings, and ongoing training and support from trained WD facilitators. The three WD facilitators were school district employees with expertise in coaching. Each facilitator supported four schools, devoting 20% of his or her time to coaching the WD teacher in each school. WD facilitators were trained during the first year of the trial by intervention team members who were part of the research staff.

**Sampling and Denominator for the Effectiveness Trial**

***Children*** The protocols for school and teacher assignment were described in the study protocol. We created protocols for student assignment as well. Each year for 3 years, all children entering first grade in each of the 12 schools were randomly assigned to classrooms on the basis of computer-generated sequences. In two prior trials conducted in Baltimore, we found that intervention impact varied by gender. Thus, in the WD trial we deliberately balanced the classrooms on gender during the random assignment process. We also created a protocol to ensure that the number of students was balanced across classrooms within a school because mobility might otherwise create an imbalance in the number of students per classroom in schools.

Random assignment of students, stratified by gender and adjusted for overall class size, began in August near the start of each school year to minimize the number of children who were randomized and then transferred out of the design. Initial random assignment was completed by research staff, and class lists were distributed to the schools. Because the intervention occurred over the course of first grade, we decided to randomly assign any children who enrolled over the course of the entire school year because they would receive a “dose” of the intervention. To protect against the differential assignments that might occur if school staff assigned children to classrooms based on their knowledge of the interventions and to minimize the burden to school staff, we developed an easy-to-use sealed-envelope system, which employed the same computer-generated sequencing used in making the initial assignments.

Each school received two stacks of numbered envelopes: a stack of envelopes for girls and a stack of envelopes for boys. School office staff kept up-to-date class lists for each first-grade class. Upon enrolling a new first-grade student, school staff opened the first envelope from either the girls’ or boys’ pile, depending on the gender of the newly enrolled student. To ensure that classrooms remained balanced with regard to the number of students, a sheet with simple instructions began with the following question: “If this student gets assigned to the teacher named above, will she have 4 more students in her class than another first-grade teacher?” If the answer to the question was yes, the directions were (1) Check the box marked “Yes,” (2) Fax the form to the research office, and (3) Open the next envelope. If the answer was no, the directions were (1) Assign the student to the teacher named above; (2) Complete information about the student including name, gender, date of birth; and (3) Fax the form to the research office. School office staff were trained to call research staff whenever a student could not be assigned according to the predetermined instructions outlined in the gender-specific envelopes. The most common reason school staff and research staff conferred about assignment were instances in which random assignment would have resulted in siblings or cousins being assigned to the same classroom.

With continued entrances and exits of children in classrooms throughout the year, we needed to specify exactly who was to be included in intent-to-treat analyses in this study. Brown and colleagues [8] discuss appropriate ways to define the denominator at the individual level in a multilevel trial such as the WD trial. Two factors informed our decision with regard to the denominator for intent-to-treat analyses and for follow-up. First, the intervention was delivered throughout the entire school year; second, we had conducted random assignment whenever a child entered a study school through the entire school year. We determined the denominator to be all those children who were in one of the 24 WD or SC classrooms at the time of the baseline assessments in the fall of first grade prior to the beginning of the intervention *and* all children who were randomly assigned to one of the 24 classrooms throughout the school year.

Given our definition of the denominator, it is important to know the entrances and exits of individuals into the trial with regard to dosage of intervention as well as possible changes in design status. Figure 1 shows the assignment of the cohort 2 students over the entire period of random assignment: the initial period of assignment that ran from mid-August through the fielding of the baseline assessments and the postbaseline period that ran from baseline assessments to the end of the school year. Changes in study condition are noted on the figure. Children might have changed their study condition for two reasons. Children were sometimes reassigned to other classrooms/conditions with the same school. For example, in school 6, children from two classrooms were randomly chosen to create a third classroom. We called these changes administrative design changes. The second reason children changed design status was that *no shows*—children randomly assigned to a study condition between mid-August who left the school before the intervention began—sometimes transferred to other WD schools. When this occurred, the students were randomly assigned at the new school in accordance with the envelope method. Students who had a change in design status are identified twice on Figure 1. They are identified in a circle when leaving a study condition and in a triangle when reassigned to a study condition. These students are counted only once in the denominator, under the condition to which they were reassigned.

To illustrate using Figure 1, in mid-August, 342 children were assigned to the WD condition in the 12 trial schools, 276 began first grade in a WD classroom, and 66 did not show up to one of the 12 trial schools at the beginning of first grade and hence did not receive any exposure to the intervention condition in these classes. Of the 66 WD no shows, 3 transferred into other schools in the trial before the end of the baseline assessments; 1 of the 3 was reassigned to the WD condition and 2 were reassigned into the SC condition. Two more re-enrolled in trial schools during the school year after the intervention began; 1 was reassigned to WD and 1 was reassigned to a classroom not in the trial. Of the 276 students initially assigned to WD who began first grade in a WD classroom, 11 children were reassigned because of an administrative design change from WD to SC within the same school. Finally, between mid-August and the end of the baseline assessments, prior to the start of the intervention, 7 children transferred into a different trial school and were assigned to WD classrooms. Two of the 7 had originally been assigned to the SC condition and were reassigned to WD because of an administrative design change; 2 were originally SC no shows and were reassigned in a new school to WD; and 3 had originally been assigned to classrooms not in the trial design.

With regard to determining the denominator, five mutually exclusive categories of individuals for the WD trial were identified by Brown and colleagues [8]: (1) *Completers*, children who were assigned to one of the intervention conditions before the intervention began and remained in the same condition to the end of first grade; (2) *Program dropouts*, children who were assigned to one of the intervention conditions before the intervention began but left the condition after the start of the intervention but before the end of first grade; (3) *Late entrants*, children who enrolled in first grade, were randomly assigned to a study condition after the intervention began, and remained in the condition to the end of first grade; (4) *Late entrants/program dropouts*, children who enrolled in first grade, were randomly assigned to a study condition after the intervention began but left the study condition before the end of first grade; and (5) *No shows*, children who were assigned to a study condition in the initial period of random assignment from mid-August to the baseline assessments but left the study condition before the beginning of the intervention. Because the *no shows* do not provide meaningful information to the study of WD impact, having left the classroom prior to the start of the intervention, we do not include them in our denominator unless they enrolled in another trial school during the year and were reassigned to a WD or an SC classroom in keeping with the protocol described above. The remaining categories of children—*completers, program dropouts, late entrants,* and *late entrants/program dropouts*—all contribute to our understanding of WD impact because the children were students during the first-grade year during the time the intervention was being delivered. The denominator for our intent-to-treat analyses and for follow-up are the 281 students exposed to WD [241 (86%) completers; 32 (11%) program dropouts; and 8 (3%) late entrants] and 299 students in SC classrooms who serve as the comparison [229 (77%) completers, 39 (13%) program dropouts; 30 (10%) late entrants; and 1 (0%) late entrant/program dropouts], a total of 580 students.

**Measures of Student Outcomes and of Context**

***Measures of student outcomes*** We studied three student outcomes: academic development, student behavior, and psychological well-being.

Academic development Both *Word Reading Skills* [9] and *The Word Reading List* [9,10] have been subjected to extensive IRT modeling and related psychometric work. To assess academic development, we used the following norm-referenced achievement measures, each a subtest of the *Woodcock Johnson III* [11]: Letter-Word Identification, Reading Fluency, Passage Comprehension, Math Fluency, Calculation, and Applied Problems.

Student behavior Timed observations of individual children’s on-task/off-task behavior and other maladaptive behaviors were collected during classroom observations using a variation of *Teacher Observation/Student Engagement* [12]. In this schema, a different child is observed each minute until all children in the classroom are observed. Within a minute’s observation, each student is rated three times for 10 seconds to determine whether she or he is on-task or off-task and exhibiting disruptive behavior, aggression, or social isolation. Approximately 5 to 7 minutes of data were collected on each student during a 2-hour observation period. The *Teacher Observation of Classroom Adaptation-Revised (TOCA-R)* [13] is a multi-item measure of each child’s adequacy of performance on the core tasks in the classroom as defined by teachers. The interviewer records the teacher’s ratings of the adequacy of each child’s performance on a 6-point scale (“almost never” to “almost always”) on three basic tasks: accepting authority, social participation in class, and attending/concentration. Test-retest correlations over a 4-month interval with different interviewers were .60 or higher for each of the TOCA-R subscales; the alphas were .80 or higher for each subscale. With regard to predictive and construct validity, first-grade TOCA-R teacher ratings have demonstrated continuity through adolescence and into early adulthood, most recently to age 32, predicting continuity in aggression as well as risk for illicit drug use and other problem outcomes [14].

Psychological well-being We used *Baltimore How I Feel (BHIF)*, an individually administered self-report of depressive and anxious symptoms. Children report the frequency of symptoms over the previous 2 weeks [15,16]. The alphas for the depression and anxiety items were between .79 and .85 over the course of our work. The 2-week test-retest reliability coefficients ranged from .60 in first grade to .70 in middle school for the first-generation Baltimore cohorts. The depression measures in first grade have been found to be significantly related to achievement test scores and to reading gain over the first-grade year [17,18] and into middle school. At the end of administering the skills battery to each student, the observer used *direct clinical observation (DCO)* to rate the child’s flatness, depression, anxiety, hyperkinesis, and bizarre behavior, plus a global scale of overall psychopathology. In previous research with this instrument, inter-rater agreement rose dramatically beyond mild levels of symptoms from 27–55% to 80–100% for moderate/severely symptomatic children [19]. However, even “mild” ratings proved to be predictive of psychiatric symptoms in 10-year follow-ups, especially for girls [20].

Reference List

1. Evertson CM: **Training teachers in classroom management: an experimental study in secondary school classrooms.** *Journal of Educational Research* 1985, **79:**51-58.

2. Evertson CM: **Creating conditions for learning: from research to practice.** *Theory into Practice* 1987, **26:**44-50.

3. Evertson CM: **Improving elementary classroom management: a school-based training program for beginning the year.** *Journal of Educational Research* 1989, **83:**82-90.

4. Evertson CM, Emmer ET, Sanford JP, Clements BS: **Improving classroom management: an experiment in elementary school classrooms.** *Elementary School Journal* 1983, **84:**173-188.

5. Open Court: *Framework for Effective Teaching and Learning, Grade 1: Collections for Young Scholars*. Santa Rose, CA: SRA/McGraw-Hill; 1999.

6. Barrish HH, Saunders M, Wolf MM: **Good behavior game: effects of individual contingencies for group consequences on disruptive behavior in a classroom.** *J Appl Behav Anal* 1969, **2:**119-124.

7. Reid JB, Eddy JM, Fetrow RA, Stoolmiller M: **Description and immediate impacts of a preventive intervention for conduct problems.** *Am J Community Psychol* 1999, **27:**483-517.

8. Brown CH, Liao J: **Principles for designing randomized preventive trials in mental health: an emerging developmental epidemiology paradigm.** *Am J Community Psychol* 1999, **27:**673-710.

9. Foorman BR, Francis DJ, Fletcher JM, Lynn A: **Relation of phonological and orthographic processing to early reading: comparing two approaches to regression-based, reading-level-match design.** *Journal of Educational Psychology* 1996, **14:**649-655.

10. Foorman BR, Francis DJ, Fletcher JM, Schatschneider C, Mehta P: **The role of instruction in learning to read: preventing reading failure in at-risk children.** *Journal of Educational Psychology* 1998, **90:**37-55.

11. Woodcock RW, McGraw KS, Mather N: *Woodcock-Johnson III*. Itasca, IL: Riverside Publishing; 2001.

12. Foorman BR, Schatschneider C: **Measuring teaching practice during reading/language arts instruction and its relation to student achievement.** In *Systems for Observing Teaching and Learning* . Edited by Vaughn S, Briggs KL. Baltimore: Brooks Publishing; 2003:1-30.

13. Werthamer-Larsson L, Kellam S, Wheeler L: **Effect of first-grade classroom environment on shy behavior, aggressive behavior, and concentration problems.** *Am J Community Psychol* 1991, **19:**585-602.

14. Ensminger ME, Juon HS, Fothergill KE: **Childhood and adolescent antecedents of substance use in adulthood.** *Addiction* 2002, **97:**833-844.

15. Ialongo NS, Werthamer L, Kellam SG, Brown CH, Wang S, Lin Y: **Proximal impact of two first-grade preventive interventions on the early risk behaviors for later substance abuse, depression, and antisocial behavior.** *Am J Community Psychol* 1999, **27:**599-641.

16. Ialongo NS, Poduska JM, Werthamer L, Kellam S: **The distal impact of two first-grade preventive interventions on conduct problems and disorder in early adolescence.** *Journal of Emotional and Behavioral Disorders* 2001, **9:**146-160.

17. Kellam SG, Mayer LS, Rebok GW, Hawkins WE: **The effects of improving achievement on aggressive behavior and of improving aggressive behavior on achievement through two prevention interventions: an investigation of causal paths.** In *Adversity, Stress and Psychopathology*. Edited by Dohrenwend B. New York: Oxford University Press; 1998:486-505.

18. Kellam SG, Rebok GW, Ialongo N, Mayer LS: **The course and malleability of aggressive behavior from early first grade into middle school: results of a developmental epidemiologically-based preventive trial.** *J Child Psychol Psychiatry* 1994, **35:**259-281.

19. Kellam SG, Branch JD, Agrawal KC, Ensminger ME: *Mental Health and Going to School: The Woodlawn Program of Assessment, Early Intervention, and Evaluation*. Chicago: University of Chicago Press; 1975.

20. Kellam SG, Brown CH, Rubin BR, Ensminger ME: **Paths leading to teenage psychiatric symptoms and substance abuse: developmental epidemiological studies in Woodlawn.** In *Childhood Psychopathology and Development*. Edited by Guze SB, Earls FJ, Barrett JE. New York: Raven Press; 1983:17-51.
