# Supplementary material for: Study protocol for a group randomized controlled trial of a classroom-based intervention aimed at preventing early risk factors for drug abuse: integrating effectiveness and implementation research
Source: Implement Sci. 2009 Sep 2;4:56. doi: 10.1186/1748-5908-4-56 (PMC2753630; doi:10.1186/1748-5908-4-56)
Supplement: Additional file 2 — WD cohort 2 student sample. This figure shows the random assignment and design status of a cohort of students from initial random assignment prior to the start of first grade to the end of the first grade year. WD = students randomized to Whole Day First Grade Program; SC = students randomized to standard classroom (control); Other = students randomized to first grade classrooms not participating in the trial; circles denote students who left a study condition and were reassigned to another study condition (denoted by a triangle). [file 1748-5908-4-56-S2.pdf]

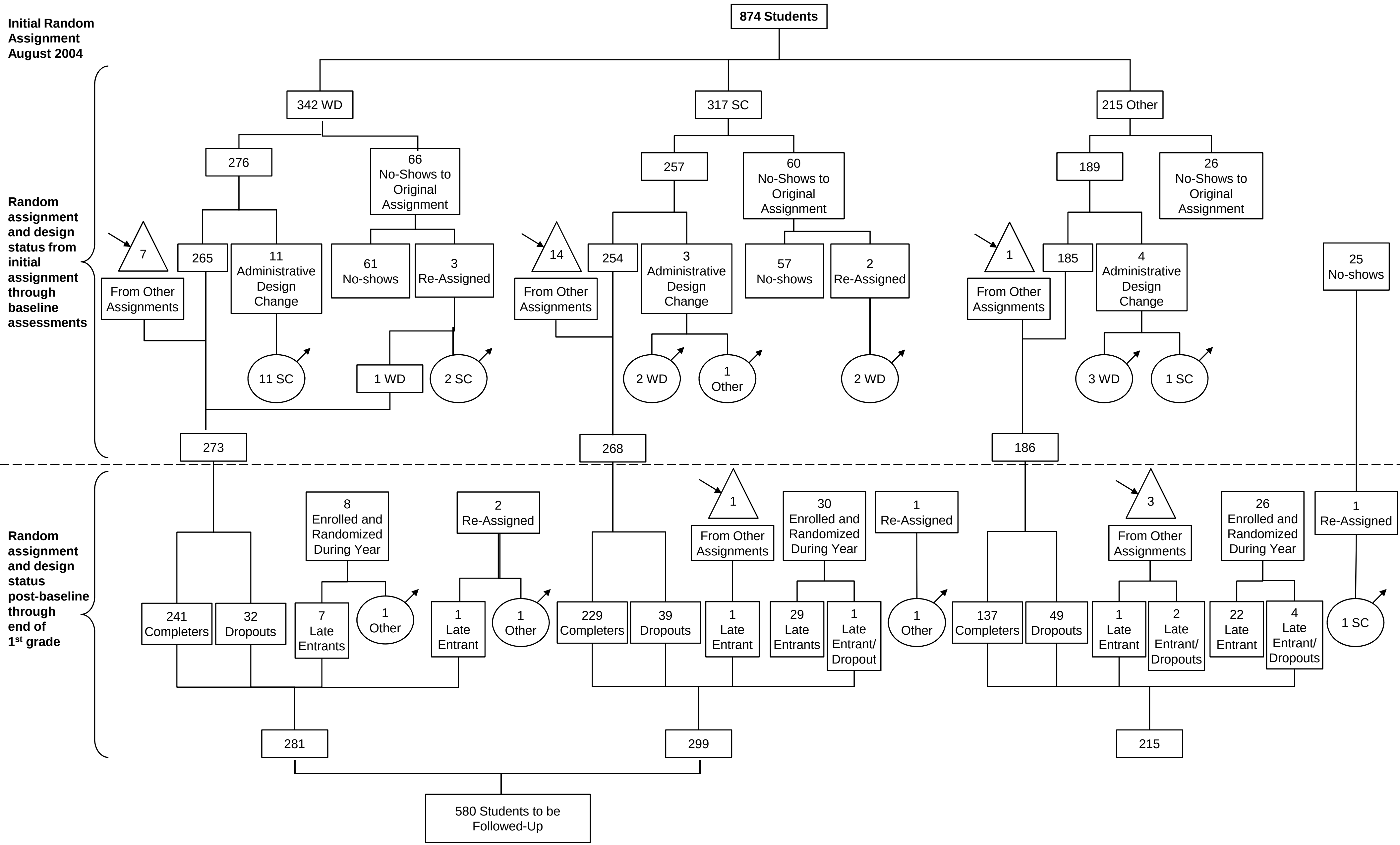
Initial Random Assignment August 2004
874 Students
342 WD
317 SC
215 Other
Random assignment and design status from initial assignment through baseline assessments
276
66 No-Shows to Original Assignment
257
60 No-Shows to Original Assignment
189
26 No-Shows to Original Assignment
7 From Other Assignments
265
11 Administrative Design Change
61 No-shows
3 Re-Assigned
14 From Other Assignments
254
3 Administrative Design Change
57 No-shows
2 Re-Assigned
1 From Other Assignments
185
4 Administrative Design Change
25 No-shows
11 SC
1 WD
2 SC
2 WD
1 Other
2 WD
3 WD
1 SC
273
268
186
Random assignment and design status post-baseline through end of 1st grade
8 Enrolled and Randomized During Year
2 Re-Assigned
1 From Other Assignments
30 Enrolled and Randomized During Year
1 Re-Assigned
3 From Other Assignments
26 Enrolled and Randomized During Year
1 Re-Assigned
241 Completers
32 Dropouts
7 Late Entrants
1 Other
1 Late Entrant
1 Other
229 Completers
39 Dropouts
1 Late Entrant
29 Late Entrants
1 Late Entrant/ Dropout
1 Other
137 Completers
49 Dropouts
1 Late Entrant
2 Late Entrant/ Dropouts
22 Late Entrant
4 Late Entrant/ Dropouts
1 SC
281
299
215
580 Students to be Followed-Up
